# Sustained Treatment with Insulin Detemir in Mice Alters Brain Activity and Locomotion

**DOI:** 10.1371/journal.pone.0162124

**Published:** 2016-09-02

**Authors:** Tina Sartorius, Anita M. Hennige, Andreas Fritsche, Hans-Ulrich Häring

**Affiliations:** 1 Department of Internal Medicine, Division of Endocrinology, Diabetology, Vascular Disease, Nephrology and Clinical Chemistry, University of Tuebingen, Tuebingen, Germany; 2 German Center for Diabetes Research (DZD), Tuebingen, Germany; 3 Institute for Diabetes Research and Metabolic Diseases of the Helmholtz Center Munich at the University of Tuebingen (IDM), Tuebingen, Germany; Hospital Infantil Universitario Nino Jesus, SPAIN

## Abstract

**Aims:**

Recent studies have identified unique brain effects of insulin detemir (Levemir^®^). Due to its pharmacologic properties, insulin detemir may reach higher concentrations in the brain than regular insulin. This might explain the observed increased brain stimulation after acute insulin detemir application but it remained unclear whether chronic insulin detemir treatment causes alterations in brain activity as a consequence of overstimulation.

**Methods:**

In mice, we examined insulin detemir’s prolonged brain exposure by continuous subcutaneous (s.c.) application using either micro-osmotic pumps or daily s.c. injections and performed continuous radiotelemetric electrocorticography and locomotion recordings.

**Results:**

Acute intracerebroventricular injection of insulin detemir activated cortical and locomotor activity significantly more than regular insulin in equimolar doses (0.94 and 5.63 mU in total), suggesting an enhanced acute impact on brain networks. However, given continuously s.c., insulin detemir significantly reduced cortical activity (theta: 21.3±6.1% vs. 73.0±8.1%, *P*<0.001) and failed to maintain locomotion, while regular insulin resulted in an increase of both parameters.

**Conclusions:**

The data suggest that permanently-increased insulin detemir levels in the brain convert its hyperstimulatory effects and finally mediate impairments in brain activity and locomotion. This observation might be considered when human studies with insulin detemir are designed to target the brain in order to optimize treatment regimens.

## Introduction

Recent trials have indicated that insulin detemir exerts unique brain effects [1–3]. It is believed that insulin detemir’s weight-sparing effect might be ascribed to an increased action in the central nervous system (CNS) [2]. Human studies further revealed that insulin detemir overcomes cerebrocortical insulin resistance in obese subjects [4], and acutely intravenously applied insulin detemir induced changes in cortical activity in men [5]. Therefore, insulin detemir is supposed to acutely activate brain insulin signalling more than regular human insulin, and even overcomes insulin resistance in obese subjects, possibly due to acutely raising brain insulin concentrations. However, as most of the studies demonstrated insulin detemir’s brain efficacy when applied acutely [5, 6], it is still unknown whether these CNS effects will prevail in the long-run or whether undesired effects due to hyperinsulinemia in the brain will become apparent.

From peripheral tissues it is known that mild hyperinsulinemia is well-tolerated in the short-term to overcome insulin resistance but permanent hyperinsulinemia contributes to insulin resistance in obesity and type-2-diabetes. Regarding chronic insulin detemir therapy, however, it remains to be determined whether permanent hyperinsulinemia might in turn cause brain insulin resistance. Therefore, the impact of elevated brain insulin concentrations has to be assessed to judge the consequences of lifelong insulin detemir therapy. Application regimens optimization is required to efficiently overcome pre-existing brain insulin resistance in obese individuals and intranasal insulin delivery is attracting increasing attention as alternative treatment option [7]. First evidence that this concept might be promising came from a recent study showing improved cognition in adults diagnosed with Alzheimer’s disease in a 3-week-protocol [1].

The current experiments were designed to test whether insulin detemir may affect brain activity and locomotion, which might counteract the desired effect of insulin detemir brain therapy in humans. Here, we focused on the characterization of effects in the brain due to hyperinsulinemia and compared regular insulin and insulin detemir in equimolar and equipotent concentrations.

## Materials and Methods

### Animals

10 to 15-week aged male C57BL/6NCrl mice were purchased from Charles River Laboratories (Sulzfeld, Germany) and were maintained on a 12-h light-dark cycle (lights on from 7 a.m. to 7 p.m.) with free access to water and a mouse laboratory chow (Diet#1310, Altromin, Lage, Germany) and with all appropriate guidelines through the University of Tuebingen. All procedures were approved by the University of Tuebingen Institutional Animal Care and Use Committee (Permit Number M1/06). All surgery was performed under ketamine/xylazine anaesthesia combined with isoflurane/oxygen gas anaesthesia (1.5%; mixed with 4 L/min oxygen). Anesthesia depth was determined by respiration rate, vibrissae movements, and eyelid reflex. Body temperature was monitored by a thermistor placed underneath the mouse’s abdomen and maintained at 37°C using a heating pad. For pre-emptive analgesia we subcutaneously administered Carprofen (Rimadyl®, 5 mg/kg) intraoperative and further provided it once daily for the consecutive two post-surgical days. After surgery, animals were individually housed in cages containing nesting material, and a recovery period of 8 days was awaited before starting the experimental measurements. The recovery of mice was carefully monitored twice per day with specific score sheets that defined experimental endpoints. For instance, the clinical signs used to determine such endpoints were loss in body weight of >20% compared to pre-surgical body weight, inappetence, apathy, torticollis, or opisthotonus. Moreover, further euthanasia was defined in terms of wound healing dysfunction or brain trauma (apparent by ECoG signal differences). All animals recovered well and thus, euthanasia prior to the experimental endpoint did not occur. During the experimental procedure animals were monitored at least three times per day. Animal sacrifice was humanely performed by cervical dislocation for adult mice.

### Intracerebroventricular (i.c.v.) Injection, Electrocortico-graphy (ECoG) and Locomotor Activity in Mice

Each mouse received a radiotelemetry ECoG transmitter and i.c.v. cannula for microinjection of substances into the lateral ventricles as previously described [8, 9]. Therefore, after transmitter implantation, a sterile 27 G stainless steel cannula 6 mm in length was inserted in the left lateral ventricle with the following coordinates: 0.3 mm posterior and 1 mm lateral relative to bregma (left hemisphere), and 3.0 mm down the skull surface. The cannula was fixed in place with dental acrylic cement together with electrodes and micro-screws. A tubing dummy prevented blockage of the cannula. The correct position of the i.c.v. cannula was verified by dye injection into the ventricles at the end of the experiments before animals were killed. Fasted mice were injected by connecting the cannula *via* a polyethylene catheter to a microinjector (CMA/Microdialysis, Solna, Sweden). Compounds injected intracerebroventricularly were freshly prepared, dissolved in sterile saline solution and delivered in a volume of 2 μL over 1 minute. Mice received equimolar i.c.v. injections of low- or high-dosed regular insulin (3.75/ 22.5 mU in total) or insulin detemir (0.94/ 5.63 mU in total), or vehicle solution (0.9% NaCl) in random order 4 days apart. After administration, telemetry signals (ECoG, locomotion) were recorded continuously for 120 min, processed by a Data-Sciences analogue converter (Data Exchange Matrix, DSI, St. Paul, MN, USA) and stored digitally using the Dataquest A.R.T. 3.1 software (DSI). This software coordinates the detection and collection of continuously assessed signals in real-time from the animals residing in their own familiar environment [10]. Using fast Fourier transformation for theta (4–8 Hz), alpha (8–12 Hz), and beta (12–30 Hz) frequency bands, the power spectral density was estimated and displayed as percent change of vehicle application (baseline: 0%) to exclude inter-individual effects. Locomotor activity was continuously measured by detecting changes in signal strength that occurred as the animals moved about their cages. Changes in signal strength generated a digital pulse, which was counted by the data-acquisition system [10, 11]. Thus, locomotor activity was recorded continuously and stored at 1 minute intervals.

### Micro-Osmotic Pump Implantation and Metabolic Characterization

Micro-osmotic pumps (ALZET, CA, USA) were subcutaneously implanted. The pumps were primed *in vitro* prior to *in vivo* implantation by equilibration the filled pumps in sterile 0.9% saline at 37°C overnight. During the 8-day period, mice received continuous administration of 0.25 μL/h (equals to 0.6 U/d) of regular insulin, insulin detemir or saline as control. Glucose levels were assessed in the morning using a glucometer (Bayer, Leverkusen, Germany). Serum insulin/glucagon levels were measured by RIA (Millipore, Schwalbach, Germany).

### Intermittent low-dosed subcutaneous (s.c.) injections

After assessing cortical and locomotor activity under basal, untreated control condition for 3 days, mice were subcutaneously injected with freshly-prepared equipotent doses (equals to half of the concentration used *via* micro-osmotic pumps) of regular insulin or detemir once- (7 a.m.; 0.3 U/ 100 μL) or twice-daily (7 a.m./ 7 p.m.; 0.15 U/ 100 μL). During the 3-day treatment period, blood glucose levels were assessed before s.c. morning injections and cortical and locomotor activity were continuously determined by radiotelemetry.

### Data Analysis and Statistics

ECoG data analysis was performed as previously described [8]. Data were analysed using Origin 8.1. Significance for all analyses: *P*<0.05. Comparisons between groups were analysed by one-way or two-way ANOVA when appropriate followed by Bonferroni’s (compares only selected pairs of means) or Tukey’s (means comparison method) post-hoc comparisons.

## Results

**Fig 1:**
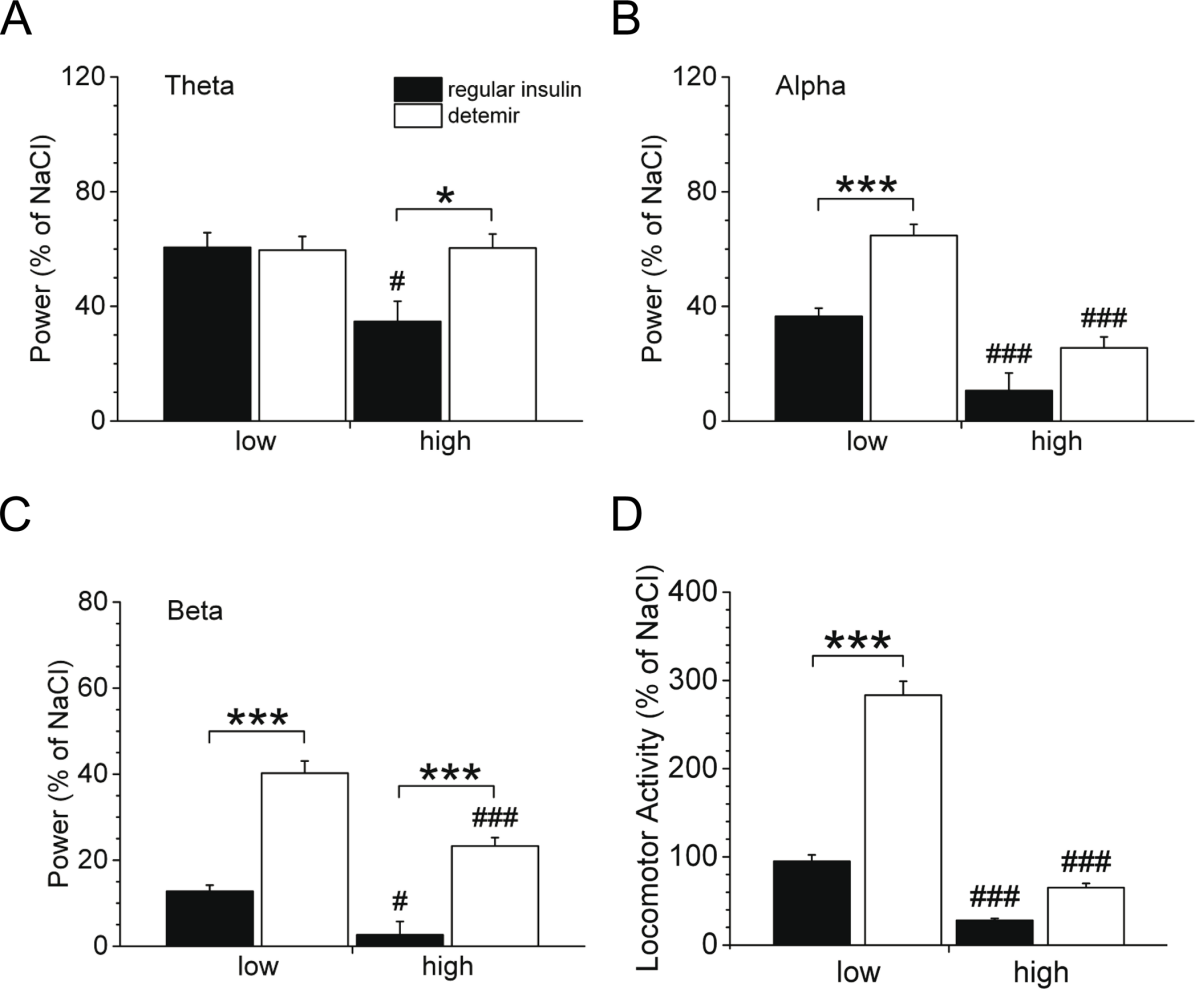
Cortical and locomotor activity following intracerebroventricular (i.c.v.) injection. Mice were injected i.c.v. with a single bolus of either regular insulin or insulin detemir in low or high concentrations (please see method section for details). **(A-C)** Power spectral density (expressed as % of the saline experiment) of **(A)** theta, **(B)** alpha and **(C)** beta frequency bands for the 120-min post-injection period. **(D)** Quantification of locomotor activity (expressed as % of the saline experiment) for the 120-min post-injection period. Significance between regular insulin and insulin detemir: *P<0.05, ***P<0.001. ^#^*P*<0.05, ^###^*P*<0.001 depict significance between low and high concentrations within respective insulin group; *n* = 7-8/group. Data are mean±SEM.

We first performed studies to acutely raise brain insulin concentrations, and assessed the effect of a low or high dose of either regular insulin or insulin detemir on brain activity and locomotion. Thus, mice were injected intracerebroventricularly with either regular insulin or insulin detemir in various doses. Insulin detemir activated the theta (Fig 1A), alpha (Fig 1B), and beta (Fig 1C) frequency bands to the same (theta) or to a significantly higher (alpha, beta) degree as regular insulin when applied in equimolar low doses (Fig 1A–1C, left panel). By increasing the dose, we examined whether elevated brain insulin concentrations in turn further affect cortical and locomotor activity. Regular insulin as well as insulin detemir significantly inhibited cortical activity (Fig 1A–1C, right panel), suggesting that supraphysiologically elevated brain insulin levels provoke an impairment in insulin sensitivity as low insulin-stimulated brain activity was shown to come along with diminished insulin sensitivity [8, 9, 12–14]. Nonetheless, insulin detemir was still able to induce cortical activity more than regular insulin, and low-dosed insulin detemir significantly elevated locomotion compared to regular insulin (Fig 1D, left panel). However, both insulins were less effective to induce locomotion in higher dosages (Fig 1D, right panel).

**Fig 2:**
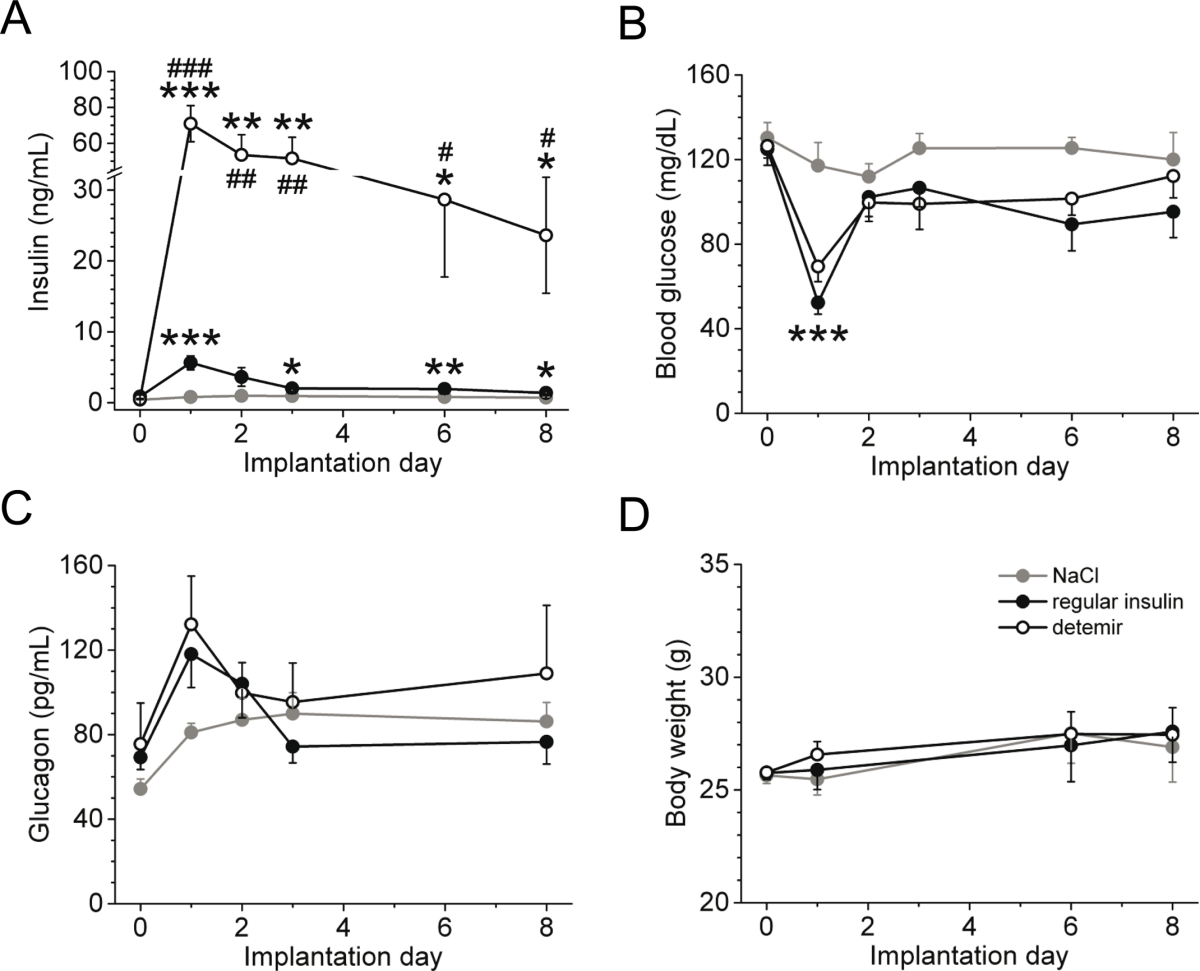
Metabolic parameters of continuously applied regular insulin or insulin detemir by using micro-osmotic pumps. **(A)** Plasma insulin levels following implantation of micro-osmotic pumps containing either regular insulin, insulin detemir or saline. **(B-D)** Blood glucose concentrations **(B)**, plasma glucagon levels **(C)** and body weight **(D)** of mice implanted with micro-osmotic pumps. Equipotent doses of regular insulin and insulin detemir were used; *n* = 5/group. *P<0.05, **P<0.005, and ***P<0.001 depict significance of both regular insulin and insulin detemir groups to the saline group. Significance between regular insulin and insulin detemir: ^#^*P*<0.05, ^##^*P*<0.005, and ^###^*P*<0.001. Data are mean±SEM.

To test whether insulin detemir alters cortical activity and locomotion by continuous treatment, we subcutaneously applied insulin for 8 days using micro-osmotic pumps. Plasma insulin concentrations were significantly elevated by insulin detemir due to its higher equipotent dose (Fig 2A), and glucagon levels significantly increased at day 1 after implantation of the insulin pumps due to relative hypoglycaemia (~60 mg/dL, Fig 2B) but did not vary between both insulins (Fig 2C). No difference in body weight was observed (Fig 2D), and food intake was indistinguishable between the groups (data not shown).

**Fig 3:**
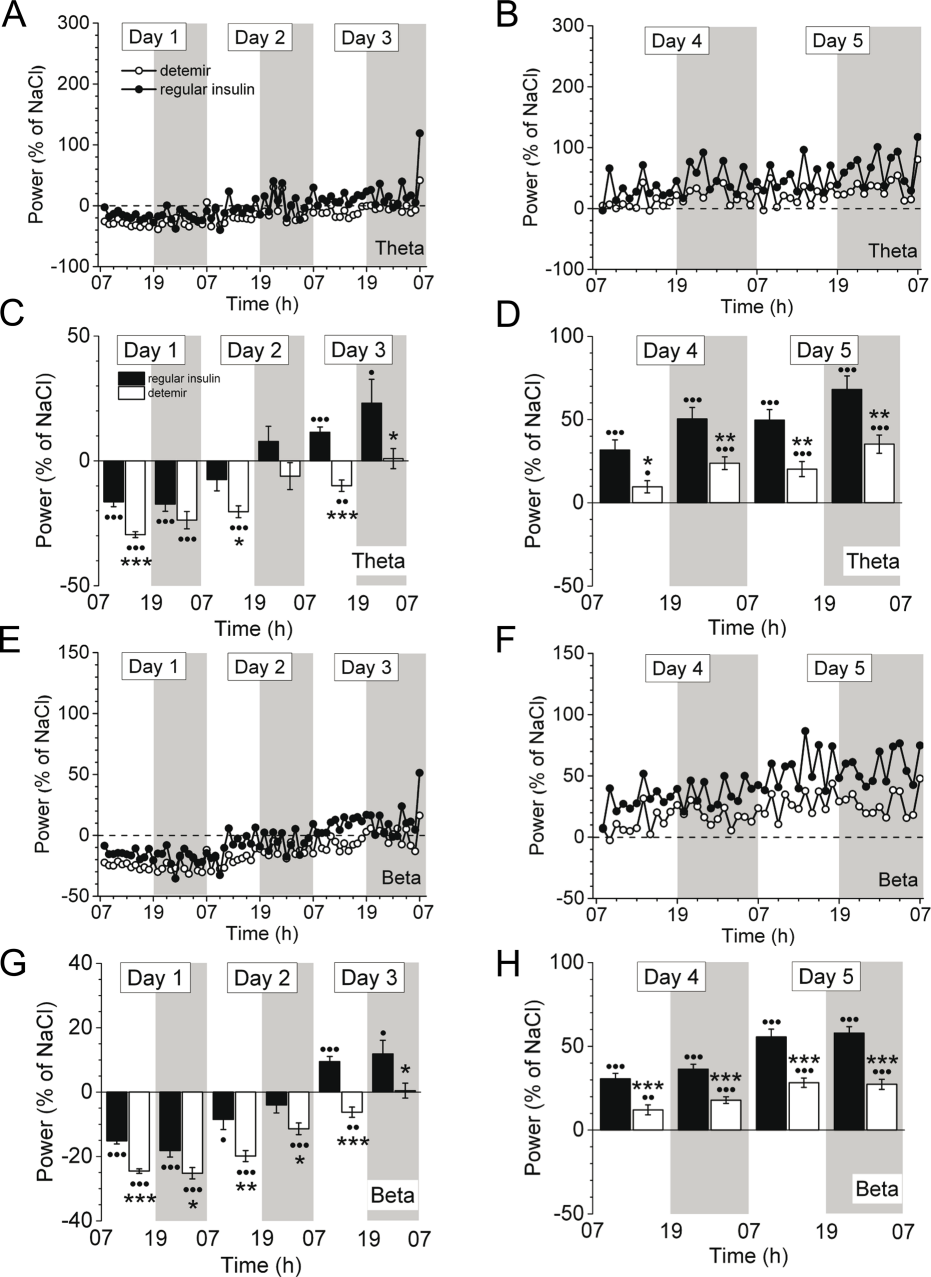
Cortical activity of mice in the early period of continuous treatment with either regular insulin or insulin detemir by using micro-osmotic pumps. Cortical activity is indicated over time **(A,B,E,F)** or as 12-h average±SEM **(C,D,G,H)** for the theta **(A-D)** and beta **(E-H)** frequency bands. Equipotent glucose-lowering doses of regular insulin and insulin detemir (both 0.6 U/d) were used. Representative data (as percentage of saline) are shown for days 1 to 3 **(A,C,E,G)** and days 4 to 5 **(B,D,F,H)** after implantation of the pumps containing either regular insulin or insulin detemir. •*P*<0.05, ••*P*<0.005, •••*P*<0.001 indicate significance to saline. Significance between treatment groups as follows: *P<0.05, **P<0.005, ***P<0.001. *N* = 5 for regular insulin and insulin detemir groups, *n* = 3 for saline group.

**Fig 4:**
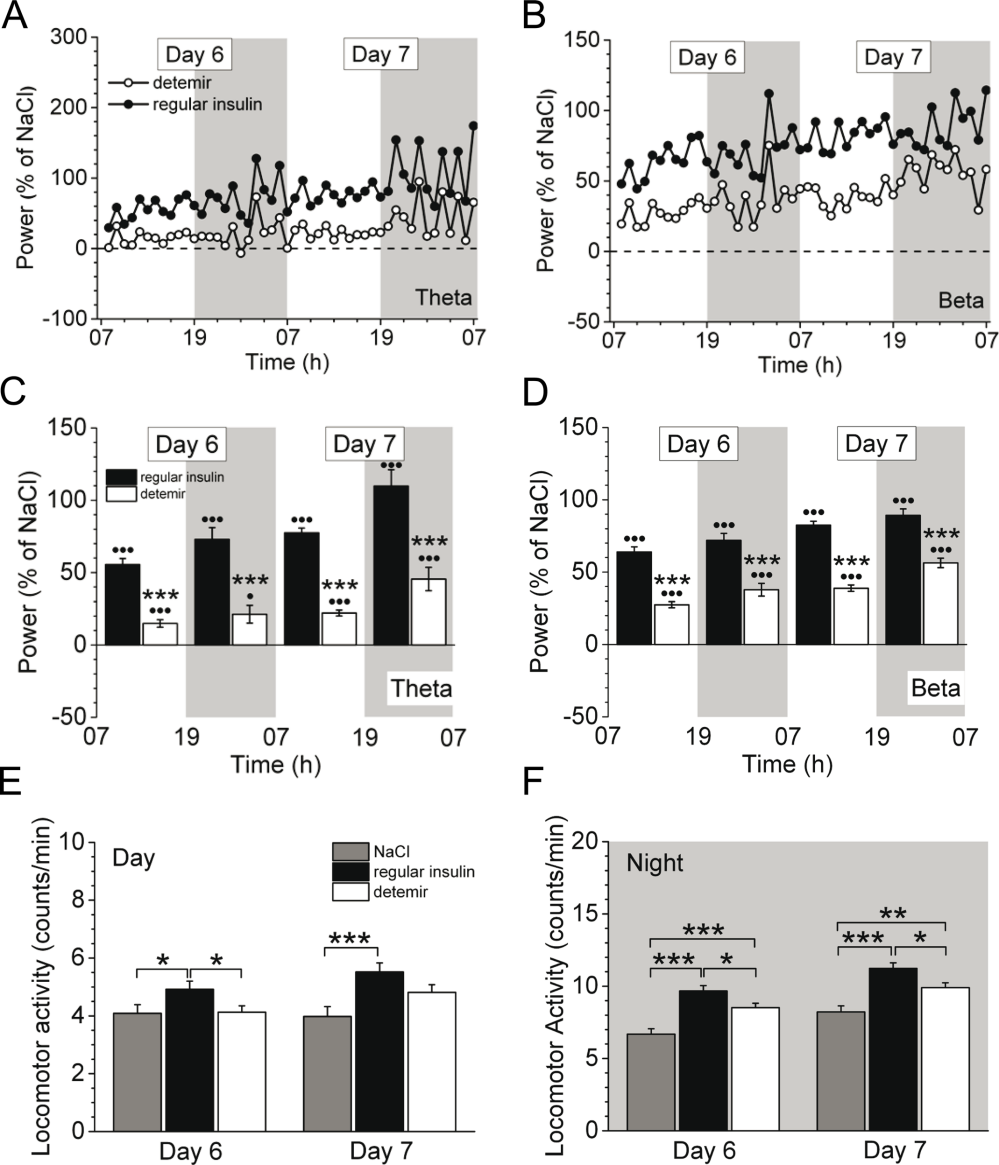
Cortical and locomotor activity in mice continuously treated with either regular insulin or insulin detemir using micro-osmotic pumps. Cortical activity is indicated over time **(A,B)** or as 12-h average±SEM **(C,D)** for the theta **(A,C)** and beta **(B,D)** frequency bands. Representative data are shown for days 6 and 7 after pump implantation containing either regular insulin or insulin detemir. •*P*<0.05, •••*P*<0.001 indicate significance to saline. **(E,F)** Quantification of locomotion for days 6 and 7 during the day **(E)** and night **(F)** periods. Significance between treatment groups as follows: *P<0.05, **P<0.005, ***P<0.001. *N* = 5 for regular insulin and insulin detemir groups, *n* = 3 for saline group.

From day 3 onwards, cortical activity in all frequency bands constantly increased for both insulins, however, it remained lower in insulin detemir-treated animals compared to regular insulin through day 5 (Fig 3A–3H). Moreover, insulin detemir activated cortical activity of theta (Fig 4A and 4C) and beta (Fig 4B and 4D) bands to a significantly lesser extent at days 6–7 post-implantation versus regular insulin (theta: 21.3 ± 6.1% *vs*. 73.0 ± 8.1%, *P*<0.001; beta: 37.8 ± 4.4% *vs*. 72.0 ± 4.8%, *P*<0.001 (day 6, night)). This was also obvious for delta and alpha frequency bands (data not shown). Locomotion was instantly increased in insulin detemir-treated mice at day 1 when insulin concentrations in the brain were acutely raised (S1 Fig), but then insulin detemir failed to increase locomotion to the same degree as regular insulin as demonstrated for days 6–7 (Fig 4E and 4F).

**Fig 5:**
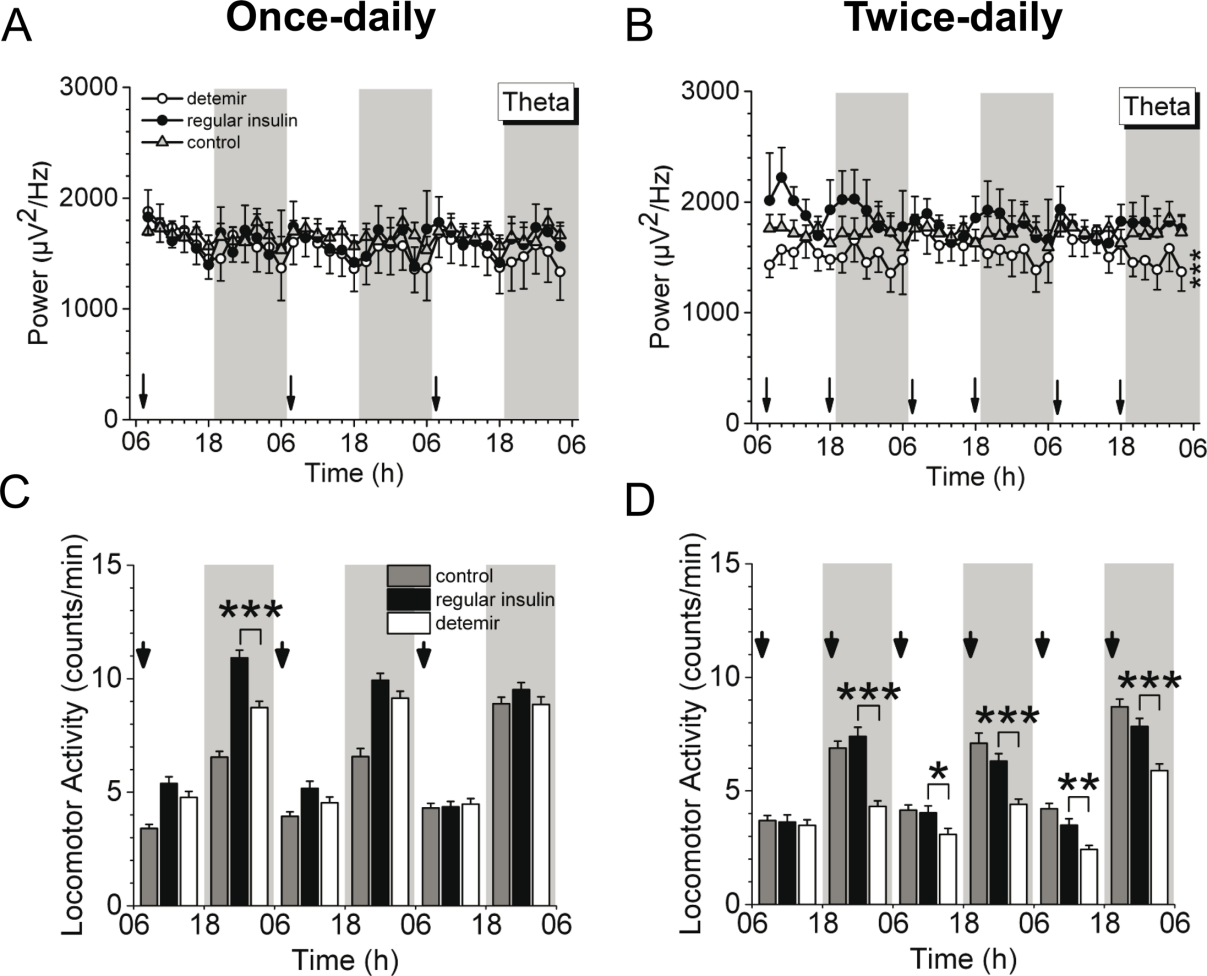
Once- *vs*. twice-daily subcutaneous treatment with regular insulin or insulin detemir. Both regular insulin and insulin detemir were subcutaneously injected either once-daily (0.3 U/injection) or twice-daily (0.15 U/injection) and compared to the basal, untreated control condition. **(A-D)** Theta activity **(A,B)** and locomotion **(C,D)** during the once- **(A,C)** or twice-daily **(B,D)** insulin injections. Data are 2-h average±SEM (for **A**,**B**) or 12-h average±SEM (for **C**,**D**). Significance between treatment groups as follows: *P<0.05, **P<0.005, ***P<0.001. *N* = 4-7/group. Arrow indicate injections.

Comparing the brain impact of peripherally applied insulin detemir and regular insulin in a more physiological dose range, equipotent doses were subcutaneously applied once- or twice-daily. These dosing regimens were previously applied in a randomised 52-week trial, and once-daily insulin detemir dosing appeared to be more advantageous by reducing body weight to a greater extent than twice-daily injections [15]. Thus, to gain more insight into brain activity in the initiation phase, we further compared these dosing regimens. Notably, twice-daily insulin detemir-treated mice displayed significantly reduced cortical activity compared to regular insulin and control (Fig 5B) while no difference was observed by once-daily injections during the 3 days-lasting treatment protocol (Fig 5A). Similar results could be revealed for locomotion: once-daily injected insulin detemir did not differ from regular insulin (Fig 5C) whereas a significant decrease was apparent with twice-daily injections and therefore shorter injection intervals (Fig 5D).

## Discussion

This study provides new insight into insulin detemir’s brain effect when applied continuously. Using different approaches we found that insulin detemir induces brain effects as demonstrated by diminished cortical activity and locomotion as behavioural readout. It is still a matter of debate if such reduced alterations point to an impaired insulin sensitivity or even insulin resistance in the brain. Of note, an impaired brain activity in the slow frequency range with reduced locomotion could be revealed in diet-induced insulin resistant mice, and this went along with brain insulin resistance on the molecular level [8]. Moreover, previous studies demonstrated that insulin-stimulated cerebrocortical theta activity is negatively correlated with increased serum concentrations of saturated nonesterified fatty acids [13], a fatty acid class commonly associated with impaired insulin sensitivity. In addition, subjects with impaired insulin sensitivity were further characterized by low insulin-stimulated beta and theta activity, and lifestyle intervention resulted in an amelioration of brain activity [12]. Thus, one might speculate that insulin detemir’s effect on CNS in the long-term may be due to its accumulation in the CSF as insulin detemir crosses the blood-brain barrier more easily due to its lipophilic property [3, 4, 16]. This is in accordance with a recent study substantiating that peripherally applied insulin detemir is transported from blood to CSF at a higher rate, and once there, remained elevated significantly longer [2]. Moreover, this might also explain why acutely applied insulin detemir induces stronger effects on brain function than human insulin [3–6], and even amplify the impact of hypoglycemia in the CNS [17]. Consistently, our results suggest that low-dosed insulin detemir enhanced cortical and locomotor activity more than regular insulin when acutely applied intracerebroventricularly. This implies that cortical activity changes are due to brain insulin action when given peripherally, and one might further speculate about a complex interplay of surface binding, surface detachment, receptor cycling (internalization and reinsertion) or stimulation of alternate pathways in the brain *in vivo*. Of note, as prolonged exposure to insulin results in receptor degradation and a net loss of total cell receptors in primary cultured adipocytes [18], one might assume similar for high-dosed detemir in the brain. In this context, one might further reason that more unbound active detemir is available to act with insulin receptors in the brain by a sustained application regime as albumin concentration in the CSF is very low compared to the blood. However, further studies are needed to reveal insulin detemir’s pharmacokinetics in the brain and the specific/preferable insulin detemir-affected brain regions having a greater impact on neuronal function, brain activity and locomotion. Regarding locomotion, this parameter is discussed to be an important behavioural readout of brain activity and insulin sensitivity, as previous studies in mice demonstrated that impaired locomotor activity goes along with insulin resistance in the periphery and the brain, also on the molecular level [8, 9, 14]. However, we cannot rule out that locomotor activity influences cerebrocortical activity or *vice versa*. Thereby, mice expressing a constitutively active version of signal transducer and activator of transcription 3 (STAT3) selectively in hypothalamic neurons of the arcuate nucleus are lean and resistant to diet-induced obesity, a phenotype arising from increased locomotor activity [19]. Furthermore, one has to take into account the counter-regulatory hormone response to insulin which might contribute to alterations of cortical and locomotor activity, for instance, *via* autonomic mechanisms. In this context, Herring and colleagues [6] have recently shown that subcutaneous insulin detemir affect the counter-regulatory hormone response to insulin which resulted in a delayed suppression of NEFA concentrations and glycerol production rate compared to NPH insulin.

So far, only two studies have reported results of chronic insulin detemir therapy in the brain [1, 20]. Adults diagnosed with Alzheimer’s disease displayed improved cognition by a 3-week intranasal insulin detemir therapy [1]. Furthermore, insulin detemir’s beneficial weight-sparing effect is partially suggested to be ascribed to an increased CNS action [2] where it reduces food intake and has a net catabolic effect in rodents [2] and humans [20]. However, despite these promising treatment options, further long-term studies have to examine the effectiveness and long-term outcome of chronic insulin detemir therapy on brain function. This might translate into a narrow therapeutic window that might hold an optimizing potential regarding weight reduction and improvement in cognition.

Due to insulin detemir’s pharmacokinetic property and its subsequent sustained elevation in the CSF, intermittent application might be the preferable regime to improve brain insulin action with its beneficial effects on brain function, locomotion and body weight, and at the same time avoid a potential “desensitization” of brain insulin receptors with subsequent signalling alterations. A negative shift of cortical theta activity and alleviated locomotion by twice-daily applications might support the fact why once-daily insulin detemir in clinical trials demonstrated weight advantage over glargine [15] while twice-daily insulin detemir therapy did not. Moreover, not only constantly-elevated insulin detemir concentrations might result in alterations of brain activity and locomotion due to a potential “desensitization” of brain insulin receptors, but also shorter injection intervals contribute to this impairment. This is of importance not least because intranasal insulin administration targeting the brain emerged as promising intervention for the treatment of cognitive impairment [1].

In summary, our findings provide insight into insulin detemir’s effect on brain activity and its relationship to locomotion when chronically applied. Because of sustained elevations of insulin CSF levels, continuous insulin detemir therapy has to be performed with caution. It is possible that a less frequent injection interval might be more appropriate to target the brain. Even so, the optimal insulin detemir dose for the purpose of positively modulate brain function and locomotion in the long-term remains unknown, and therefore, dose-response studies have to be undertaken in future experiments prioritized to brain therapy.

## Supporting Information

S1 Fig Locomotor activity of mice in the early period of continuous treatment with either regular insulin or insulin detemir by using micro-osmotic pumps. Locomotor activity is indicated as 12-h average±SEM and representative data are shown for days 1 through 5 after implantation of the pumps containing either regular insulin, insulin detemir or saline. •*P*<0.05, ••*P*<0.005, •••*P*<0.001 indicate significance to saline. Significance between treatment groups as follows: *P<0.05, ***P<0.001. *N* = 5 for regular insulin and insulin detemir groups, *n* = 3 for saline group.
(TIF)

## Abbreviations

CNS: central nervous system
CSF: cerebrospinal fluid
ECoG: electrocorticography
Hz: Hertz
i.c.v.: intracerebroventricular
s.c.: subcutaneous
