# Supplementary figures and images for: Sustained Treatment with Insulin Detemir in Mice Alters Brain Activity and Locomotion
Source: PLoS One. 2016 Sep 2;11(9):e0162124. doi: 10.1371/journal.pone.0162124 (PMC5010192; doi:10.1371/journal.pone.0162124)

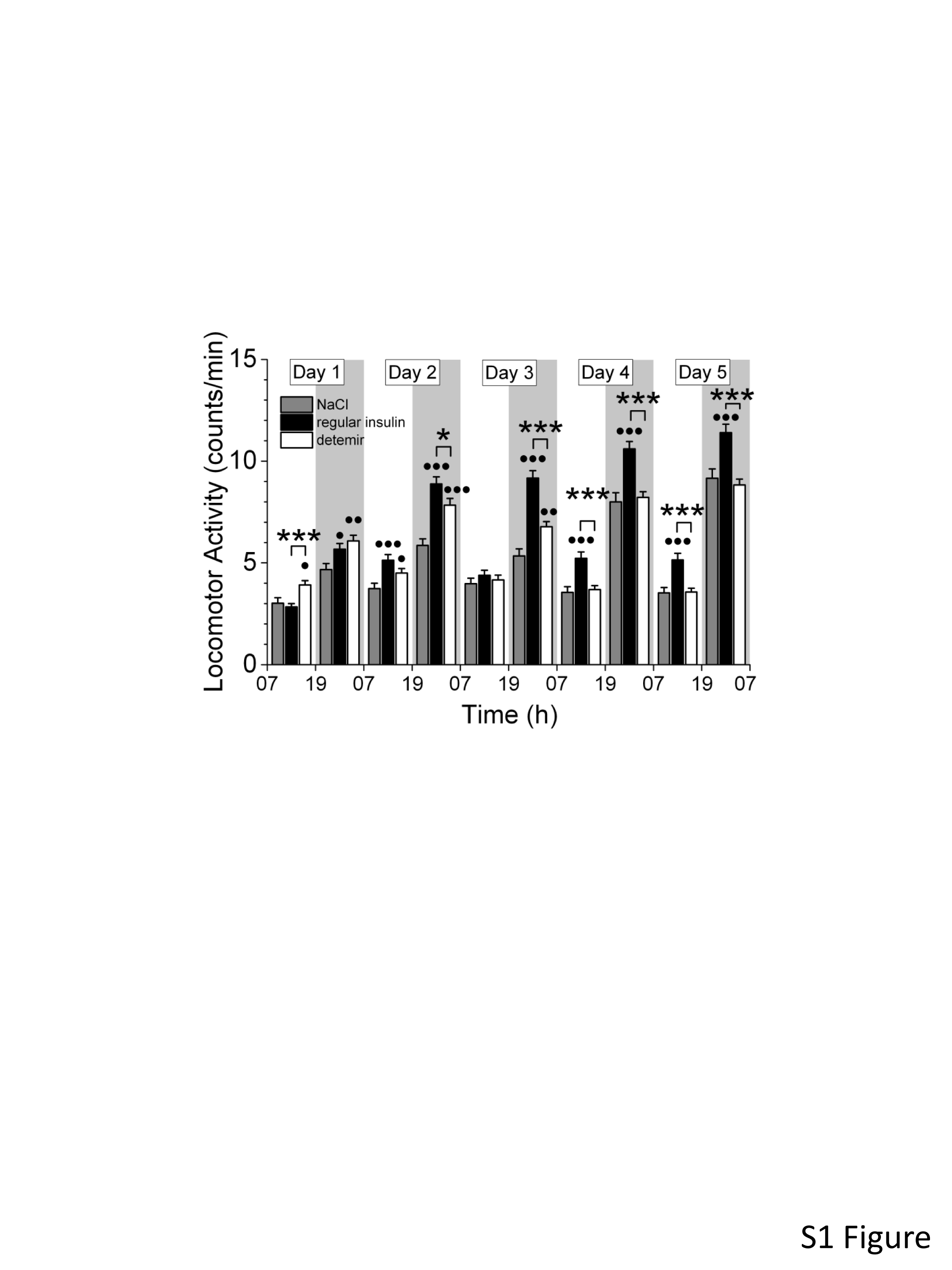

Supplement: S1 Fig — Locomotor activity is indicated as 12-h average±SEM and representative data are shown for days 1 through 5 after implantation of the pumps containing either regular insulin, insulin detemir or saline. •P<0.05, ••P<0.005, •••P<0.001 indicate significance to saline. Significance between treatment groups as follows: *P<0.05, ***P<0.001. N = 5 for regular insulin and insulin detemir groups, n = 3 for saline group. (TIF) [file pone.0162124.s001.tif]
